# Economic Evaluation of Exercise or Cognitive and Social Enrichment Activities for Improved Cognition After Stroke

**DOI:** 10.1001/jamanetworkopen.2023.45687

**Published:** 2023-11-30

**Authors:** Cassandra Adjetey, Jennifer C. Davis, Ryan S. Falck, John R. Best, Elizabeth Dao, Kim Bennett, Daria Tai, Katherine McGuire, Janice J. Eng, Ging-Yuek Robin Hsiung, Laura E. Middleton, Peter A. Hall, Min Hu, Brodie M. Sakakibara, Teresa Liu-Ambrose

**Affiliations:** 1 Faculty of Management, University of British Columbia, Kelowna, Canada; 2 Applied Health Economics Lab, University of British Columbia, Kelowna, Canada; 3 Centre for Aging SMART at Vancouver Coastal Health, Vancouver Coastal Health Research Institute, Vancouver, Canada; 4 Department of Physical Therapy, University of British Columbia, Vancouver, Canada; 5 Djavad Mowafaghian Centre for Brain Health, University of British Columbia, Vancouver, Canada; 6 Department of Kinesiology and Health Sciences, University of Waterloo, Waterloo, Canada; 7 Schlegel–UW Research Institute for Aging, Waterloo, Canada; 8 School of Public Health Sciences, University of Waterloo, Waterloo, Canada; 9 Department of Economics, Philosophy and Political Science, University of British Columbia, Kelowna, Canada; 10 Department of Occupational Science and Occupational Therapy, University of British Columbia, Vancouver, Canada; 11 Centre for Chronic Disease Prevention and Management, Southern Medical Program, The University of British Columbia, Kelowna, Canada

## Abstract

**Importance:**

Cognitive impairment is prevalent in survivors of stroke, affecting approximately 30% of individuals. Physical exercise and cognitive and social enrichment activities can enhance cognitive function in patients with chronic stroke, but their cost-effectiveness compared with a balance and tone program is uncertain.

**Objective:**

To conduct a cost-effectiveness and cost-utility analysis of multicomponent exercise or cognitive and social enrichment activities compared with a balance and tone program.

**Design, Setting, and Participants:**

This economic evaluation used a Canadian health care systems perspective and the Vitality study, a randomized clinical trial aimed at improving cognition after stroke with a 6-month intervention and a subsequent 6-month follow-up (ie, 12 months). The economic evaluation covered the duration of the Vitality trial, between June 6, 2014, and February 26, 2019. Participants were community-dwelling adults aged 55 years and older who experienced a stroke at least 12 months prior to study enrollment in the Vancouver metropolitan area, British Columbia, Canada. Data were analyzed from June 1, 2022, to March 31, 2023.

**Interventions:**

Participants were randomly assigned to twice-weekly classes for 1 of the 3 groups: multicomponent exercise program, cognitive and social enrichment activities program, or a balance and tone program (control).

**Main Outcomes and Measures:**

The primary measures for the economic evaluation included cost-effectiveness (incremental costs per mean change in cognitive function, evaluated using the Alzheimer Disease Assessment Scale–Cognitive-Plus), cost-utility (incremental cost per quality-adjusted life-year gained), intervention costs, and health care costs. Since cognitive benefits 6 months after intervention cessation were not observed in the primary randomized clinical trial, an economic evaluation at 12 months was not performed.

**Results:**

Among 120 participants (mean [SD] age, 71 [9] years; 74 [62%] male), 34 were randomized to the multicomponent exercise program, 34 were randomized to the social and cognitive enrichment activities program, and 52 were randomized to the balance and tone control program. At the end of the 6-month intervention, the cost per mean change in Alzheimer Disease Assessment Scale–Cognitive-Plus score demonstrated that exercise was more effective and costlier compared with the control group in terms of cognitive improvement with an incremental cost-effectiveness ratio of CAD −$8823. The cost per quality-adjusted life-year gained for both interventions was negligible, with exercise less costly (mean [SD] incremental cost, CAD −$32 [$258]) and cognitive and social enrichment more costly than the control group (mean [SD] incremental cost, CAD $1018 [$378]). The balance and tone program had the lowest delivery cost (CAD $777), and the exercise group had the lowest health care resource utilization (mean [SD] $1261 [$1188]) per person.

**Conclusions and Relevance:**

The findings of this economic evaluation suggest that exercise demonstrated potential for cost-effectiveness to improve cognitive function in older adults with chronic stroke during a 6-month intervention.

## Key Points

**Question:**

What is the cost-effectiveness of exercise or cognitive and social enrichment activities to improve cognition among older adults with chronic stroke?

**Findings:**

In this economic evaluation with 120 older adults from a randomized clinical trial, the multicomponent exercise program conducted over a 6-month period was cost-effective for cognitive function, with limited impact on health-related quality of life. Cognitive and social enrichment activities incurred higher costs compared with the balance and tone control group.

**Meaning:**

These findings suggest that multicomponent exercise may be a cost-effective approach to improving cognitive function in older adults with chronic stroke.

## Teaser

This economic evaluation is a cost-effectiveness and cost-utility analysis of multicomponent exercise or cognitive and social enrichment activities compared with a balance and tone program using data from a randomized clinical trial.

## Introduction

Stroke is a significant global public health concern, ranking as the second leading cause of death worldwide and a leading cause of serious disability.^1,2,3^ Despite improved acute stroke care, survivors still face challenges in physical function, cognitive abilities, and quality of life.^4,5,6^ Poststroke cognitive deficits are prevalent among older adults, affecting 21% to 38% of survivors of stroke, and are associated with increased risks of dementia, mortality, institutionalization, and functional limitations.^4,7,8^ These deficits impose substantial burden to patients, caregivers, and the health care system, resulting in a 3-fold higher economic burden for patients with stroke with cognitive impairment compared with those without cognitive deficits.^9,10,11^

While there are currently no pharmacological interventions approved for poststroke cognitive impairment, interventions, such as exercise and cognitive training, have shown promise in several studies.^12,13,14,15^ However, no randomized clinical trial (RCT) powered with a primary end point of cognition has evaluated the efficiency (ie, value for money) of these interventions, to our knowledge. Existing stroke rehabilitation economic evaluations have focused on self-management intervention,^16^ cognitive behavioral therapy,^17^ and physical fitness training,^18^ but comprehensive assessment of costs and benefits is lacking.^19^ Further research is needed in assessing the efficiency of these interventions to improve cognitive function.^20^ Hence, we conducted an economic evaluation to assess the cost-effectiveness of exercise or cognitive and social enrichment activities to improve cognitive function among patients with chronic stroke patients. Our primary goal was a cost-effectiveness analysis (incremental cost per cognitive function change) and a cost-utility analysis (incremental change per quality-adjusted life-year [QALY] gained or lost).

## Methods

This economic evaluation used data from a randomized clinical trial, approved by the University of British Columbia’s Clinical Research Ethics Board and the Vancouver Coastal Health Research Institute. Prior to participating in the study, all participants provided written and informed consent. The trial was registered at ClinicalTrials.gov (identification No. NCT01027858). We adhered to the Consolidated Health Economic Evaluation Reporting Standards (CHEERS) reporting guideline.

### Study Design

We conducted an economic evaluation alongside the Vitality study, a 3-group RCT that examined the efficacy of exercise and cognitive and social enrichment activities to promote cognitive function in individuals with chronic stroke.^21,22^ The Vitality study was a proof-of-concept RCT with a 2:2:3 allocation ratio for exercise, cognitive and social enrichment activities, and balance and tone, respectively, to enable planned contrast analysis against the control group. The allocation was concealed, and the assessors remained blinded to participants’ allocation. The trial consisted of 2 time horizons: a 6-month intervention and a 6-month follow-up study after the intervention ceased (ie, 12-month total time horizon). Randomization was carried out between June 6, 2014, and February 26, 2019.

### Participants

Details on participants recruitment were previously reported in the study protocol and the primary report.^21,22^ Briefly, the study recruited community-dwelling adults in the metropolitan area of Vancouver, Canada, who had experienced an ischemic or hemorrhagic stroke at least 12 months prior to study enrollment. Recruitment was conducted through community centers, stroke support groups, and newspapers. Participants were aged 55 years and older, had a Mini-Mental State Examination score greater than 20/30 (screening out those with moderate to severe cognitive impairment),^23^ were proficient in English, and were able to participate in a 60-minute activity with rest intervals. Individuals were excluded if they had diagnosed dementia, neurogenerative diseases affecting cognition and mobility, high risk of cardiac complications, impaired mobility, medication affecting cognition, or aphasia.

### Measures

The Vitality study collected descriptive and demographic variables, including age, body mass index, biological sex, educational status, and stroke type. Cognitive function was assessed using the Mini-Mental State Examination,^24^ and the Montreal Cognitive Assessment.^25^ Comorbidity impact was assessed with the Functional Comorbidity Index,^26^ and motor impairment levels were assessed using the Fugl Meyer Total Motor score.^27^

Secondary outcome measures included depression (assessed using the Center for Epidemiological Studies Depression scale^28^), general mobility and balance (assessed using the Short Physical Performance Battery^29^), and instrumental activities of daily living (assessed using the Lawton and Brody instrumental activities of daily living scale^30^). These are reported as baseline descriptive measures in this study.

### Intervention

The 3 interventions consisted of 60-minute classes conducted twice a week for 26 weeks. Trained instructors led the classes, maintaining a ratio of 1 instructor for every 4 participants.

#### Exercise

There were 34 participants randomized in the exercise group. Participants in the exercise group were involved in activities adapted from the community-based Fitness and Mobility Exercise program.^31^ The program included strength, aerobic, and balance training exercises, known to have positive health outcomes for survivors of stroke and older adults.^15,32^ Each class involved a warm-up, followed by specific exercises targeting different muscle groups and balance. Exercise activities progressed in intensity based on individual fitness levels, determined using the rating of perceived exertion, to ensure appropriate progression as needed.

#### Cognitive and Social Enrichment

There were 34 participants in the cognitive and social enrichment group. Participants engaged in cognitive exercises covering memory, learning, attention, and executive functions, alongside social and cognitive enrichment activities. Activities included word list memorization, brain training using a tablet application (Lumosity; Lumos Labs), and group social games.

#### Active Control

The balance and tone group included 52 participants. The program included stretches, deep breathing and relaxation techniques, general posture education, and core control exercises in the sitting position. This group served as a control for confounding variables, such as exercise received from community center visits and lifestyle changes unrelated to the study participation.

### Patient Engagement

Participants in the Vitality study were not engaged as patient partners in the study design. Interested parties, such as neurologists and physiotherapists, who actively support poststroke care were part of the study team and involved in the study design.

### Costs

Health resource utilization was monitored using self-reported questionnaires at 3, 6, and 12 months.^21^ Categories included health care professional visits, hospital admissions, and laboratory and diagnostic procedures. Costs were assigned based on standardized prices on the British Columbia Medical Services Commission Payment Schedule 2021 price list.^33^ All costs were adjusted and reported in 2022 Canadian dollars (CAD$). The cost of interventions considered program resources, trainee salary, and overhead costs, excluding research protocol costs. No discounting was applied, given the 6- and 12-month time horizons.^34^

### QALYs

QALYs were assessed using the area under the curve analysis of health state utility values obtained from the Euro-Quality of Life–3 Domains–5 Levels (EQ-5D-3L) questionnaire.^35^ The EQ-5D-3L measures quality of life across 5 domains: mobility, self-care, usual activities, pain, and depression, with 3 levels of responses for each domain.^36^ Participant responses are converted to a utility score using the Canadian valuation of EQ-5D-3L health states, ranging from −0.34 to 1, with 1 representing perfect health; 0, worst health or death; and less then 0, a state worse than death.^37^

### Effectiveness

The Alzheimer Disease Assessment Scale–Cognitive-Plus (ADAS-Cog-Plus) was the primary measure of cognitive function.^38^ This scale combines scores from the 13-item ADAS-Cog that evaluates cognitive abilities in memory, language, orientation, and executive function.^38^ In addition to the ADAS-Cog, other cognitive tests, including the Trail making tests A and B, Digit Span Forward and Backward, and Animal and Vegetable were part of the ADAS-Cog-Plus scoring.^21^ Scores on the ADAS-Cog-Plus range from −1 to 1, with a score of −1 representing healthy cognitive function; 0, minimal cognitive impairment; and 1, dementia.^38^

### Overview of Economic Evaluation

Using a Canadian health care system perspective, we conducted a cost-effectiveness and cost-utility analysis alongside the Vitality RCT.^21,22^ The economic evaluation consisted of 2 time horizons: 6 months, to reflect the cost-effectiveness of the intervention at intervention cessation, and 12 months, to reflect the cost-effectiveness of the intervention after a 6-month follow-up study. A health economics analysis plan was developed and outlined in the study protocol.^21^

### Statistical Analysis

All analyses for this economic evaluation used Stata version 17 (StataCorp). Data were analyzed from June 1, 2022, to March 31, 2023.

#### Cost-Effectiveness

The primary measure in the cost-effectiveness analysis was the incremental cost-effectiveness ratio (ICER), calculated by comparing the mean cost difference between intervention and control groups to the mean effectiveness difference, based on changes in ADAS-Cog-Plus after the 6-month exercise intervention. Although no established minimally clinically important difference (MCID) exists for the ADAS-Cog-Plus, an MCID of 3 points for the 13-item ADAS-Cog in recommended.^39^ The lack of significant efficacy evidence for cognitive and social enrichment intervention compared with balance and tone on the ADAS-Cog-Plus at the end of the intervention and follow-up, as well as for exercise compared with balance and tone at the end of the follow-up period, led to the decision not to conduct a cost-effectiveness analysis.^22^ Furthermore, the exercise and cognitive and social enrichment groups were not compared, since the Vitality study was not powered for a group comparsion.^21^

#### Cost-Utility Analysis

The outcome of the cost-utility analysis was the incremental cost-utility ratio (ICUR). The ICUR was calculated by ascertaining the mean difference in cost of providing the interventions compared with the control and dividing that by the mean difference in QALY. The difference in cost divided by the difference is utility was calculated for exercise vs balance and tone and cognitive and social enrichment vs balance and tone. A change of 0.03 QALYs, based on the EQ-5D-3L, is considered the MCID.^40^

The study used 5000 iterations of bootstrapping to estimate ICERs and ICURs with 95% CIs and plotted costs and effectiveness on a cost-effectiveness plane. The intervention’s cost-effectiveness probability was determined based on the quadrant placement.^41^ A cost-effectiveness threshold of CAD $50 000 (US $36 022.75) per QALY, based on precedent treatment in Canada, was chosen for the cost-utility analysis.^42,43^ A cost-effectiveness acceptability curve described an intervention being deemed cost-effective relative to a comparator across different willingness-to-pay thresholds (ie, the maximum values that a decision-maker is willing to pay for a health outcome).^44^

#### Base Case Analysis

The primary analysis estimated the ICERs and ICURs using the complete case data set, which included subsets for cost-effectiveness analysis (participants with complete costs and ADAS-Cog-Plus score data) and cost-utility analysis (participants with complete costs and QALY data). The number and proportion of participants with complete data and included in the analysis are presented in eTable 1 in Supplement 1.

#### Sensitivity and Subgroup Analyses

Sensitivity analysis was conducted using multiple imputations to assess uncertainty regarding missing data handling. We also conducted 3 subgroup analyses to examine the cost-effectiveness variation among participants based on literature indicating differing costs and cost-effectiveness for different subgroups: participants with a baseline ADAS-Cog-Plus score greater than 0,^11^ participants aged 80 years and older,^45^ and participants who had a hemorrhagic stroke.^46^

## Results

Among 120 participants in the Vitality study, mean (SD) baseline age was 71 (9) years, and 74 (62%) were male (Table 1). There were 34 participants randomized to the multicomponent exercise program, 34 participants randomized to the social and cognitive enrichment activities program, and 52 participants randomized to the balance and tone control program. The mean (SD) baseline score on the Montreal Cognitive Assessment was 21.9 (4.1), and the mean (SD) baseline score on the ADAS-Cog-Plus was 0.22 (0.80), indicating participants had mild cognitive impairment.

**Table 1.** Participant Characteristics at Baseline

Variable	Mean (SD)		
	Exercise (n = 34)	Cognitive and social enrichment (n = 34)	Balance and tone (n = 52)
Age, y	71 (9)	71 (9)	70 (8)
Weight, kg	75.9 (13.2)	79.9 (18.0)	77.6 (16.5)
Height, cm	166.3 (10.5)	170.2 (9.6)	166.2 (9.3)
BMI	27.4 (3.7)	27.3 (4.4)	28.0 (5.1)
Sex, No. (%)			
Male	21 (62)	23 (68)	30 (48)
Female	13 (38)	11 (32)	22 (42)
Type of stroke, No. (%)			
Hemorrhagic	8 (24)	10 (29)	15 (29)
Ischemic	22 (65)	18 (53)	33 (63)
Ischemic and lacunar	0	0	1 (2)
Lacunar	3 (9)	3 (9)	1 (2)
Unknown	1 (3)	3 (9)	2 (4)
Duration since recent stroke, mo	67.5 (48.3)	62.0 (55.6)	72.3 (57.1)
Number of strokes	1.1 (0.3)	1.2 (0.5)	1.3 (0.7)
Education >high school	32 (94)	32 (94)	47 (90)
Montreal Cognitive Assessment^a^	21.3 (3.7)	22.8 (4.0)	21.7 (4.5)
Mini-Mental State Examination^b^	27.1 (2.6)	27.6 (2.5)	27.2 (2.4)
Functional Comorbidity Index^c^	3.4 (2.0)	3.0 (1.5)	3.7 (1.8)
Center for Epidemiological Studies–Depression Scale^d^	9.6 (6.4)	8.4 (10.3)	9.9 (7.8)
Fugl Meyer Total Motor Score^e^	79.0 (40.0)	92.5 (24.0)	94.0 (9.5)
Primary clinical and economic outcome variables			
Alzheimer Disease Assessment Scale-Cognitive Plus^f^	0.39 (0.80)	0.12 (0.71)	0.17 (0.88)
EQ-5D-3L^g^	0.769 (0.125)	0.791 (0.130)	0.770 (0.106)
Secondary outcome variables			
6 min walk test, m	291.1 (147.8)	342.5 (143.4)	340.8 (131.9)
Gait speed, m/s	0.6 (0.4)	0.8 (0.4)	0.8 (0.4)
Instrumental activities of daily living^h^	6.8 (1.8)	7.0 (1.3)	6.8 (1.8)
Short physical performance battery^i^	7.6 (2.5)	8.2 (2.3)	8.8 (2.7)
Self-efficacy	1210 (485)	1270 (357.5)	1290 (497.0)

Abbreviations: BMI, body mass index (calculated as weight in kilograms divided by height in meters squared); EQ-5D-3L, Euro-Quality of Life–3 Domains–5 Levels.

^a^ Range, 0 (worst) to 30 (best); scores between 26 and 30 are considered within reference range.

^b^ Range, 0 (worst) to 30 (best); scores between 24 and 30 are considered within reference range.

^c^ Represents the number of comorbid illnesses and ranges from 0 (best) to 18 (worst); higher scores are associated with lower physical function.

^d^ Range, 0 (best) to 60 (worst).

^e^ Range, 0 (worst) to 100 (best); scores greater than 79 indicate mild motor impairment; 56 to 79, moderate impairment; 36 to 55, severe impairment; and 0 to 35, severe impairment.

^f^ The Alzheimer Disease Assessment Scale-Cognitive Plus is a measure of cognitive function ranging from −3.46 (best) to 4.31 (worst). Scores of −1 imply healthy cognitive functioning; 0, mild cognitive impairment; and 1, dementia.

^g^ Range, −0.34 (worst) to 1 (best). The EQ-5D-3L and was used to estimate health utility values.

^h^ Range, 0 (worst) to 8 (best).

^i^ Range, 0 (worst) to 12 (best); scores of 9 or less indicate increased risk for disability.

### Health Care Resource Use and Costs

Unit costs for health care cost items are presented in Table 2. The mean costs of the intervention per person was highest for participants in the cognitive and social enrichment group (CAD $1492; US $1074.24), followed by the exercise group (CAD $1090; US $784.80), then balance and tone (CAD $777; US $559.44). Participants in the cognitive and social enrichment group had a higher health care resource costs per person compared with the exercise or balance and tone groups, both at the end of intervention and during the follow-up period, in both cost-effectiveness and cost-utility analyses (Table 2). The largest health care resource cost item for all groups was health care professional visits.

**Table 2.** Unit Cost Items for Health Care Resource Utilization for Participants in the Vitality Study

Measure by program	Item cost, CAD$			
	Intervention^a^	Healthcare professional visit^b^	Hospital admission^c^	Laboratory procedures^b^
**Intervention cessation, at 6 mo**				
Cost-effectiveness analysis				
Exercise (n = 27)	1090	1032 (1016)	96 (157)	137 (217)
Cognitive and social enrichment (n = 28)	1492	1455 (1760)	84 (235)	196 (362)
Balance and tone (n = 44)	777	1079 (965)	142 (197)	162 (183)
Cost-utility analysis				
Exercise (n = 25)	1090	865 (641)	81 (131)	129 (219)
Cognitive and social enrichment (n = 23)	1492	1403 (1644)	96 (259)	220 (182)
Balance and tone (n = 44)	777	1081 (975)	151 (197)	182 (210)
**Follow-up, at 12 mo**				
Cost-effectiveness analysis				
Exercise (n = 24)	NA	2047 (2015)	154 (236)	245 (351)
Cognitive and social enrichment (n = 27)	NA	2696 (2960)	152 (310)	309 (485)
Balance and tone (n = 42)	NA	1999 (1539)	224 (285)	268 (314)
Cost-utility analysis				
Exercise (n = 20)	NA	1685 (1185)	155 (239)	227 (374)
Cognitive and social enrichment (n = 20)	NA	2810 (3114)	180 (351)	366 (525)
Balance and tone (n = 33)	NA	2170 (1685)	245 (293)	290 (336)

Abbreviation: NA, not applicable.

^a^ Estimated from Vitality study expense records.

^b^ Estimated from 2021 British Columbia Medical services plan.

^c^ Calculated from the Vancouver General Hospital fully allocated cost model.

### ADAS-Cog-Plus

Table 3 presents the ADAS-Cog-Plus scores at the end of the 6-month intervention. Mean (SD) scores were −0.192 (0.811) for the exercise group and −0.171 (0.982) for the balance and tone group, resulting in an incremental ADAS-Cog-Plus score of −0.021 (0.225). Negative scores imply better cognitive function; therefore, participants in the exercise group had better cognitive performance at the end of the intervention.

**Table 3.** Results of Cost-Effectiveness and Cost-Utility Analyses for Vitality Study at End of Intervention and Follow-Up

Measure	Intervention cessation, at 6 mo			End of follow-up, at 12 mo		
	Exercise	Cognitive and social enrichment	Balance and tone	Exercise	Cognitive and social enrichment	Balance and tone
Cost-effectiveness analysis						
Participants, No.	27	28	44	24	27	42
Cost per person, CAD$						
Mean (SD)	2351 (1188)	3220 (1973)	2163 (1150)	3529 (2260)	4630 (3253)	3273 (1826)
Difference	188 (285)	NA	0 [Reference]	NA	NA	0 [Reference ]
ADAS-Cog-Plus score						
Mean (SD)	−0.192 (0.811)	−0.184 (0.638)	−0.171 (0.982)	−0.100 (0.805)	NA	−0.254 (0.951)
Difference	−0.021 (0.225)	NA	0 [Reference]	NA	−0.192 (0.738)	0 [Reference ]
ICER	−8823^a^	NA	0 [Reference]	NA	NA	0 [Reference]
Cost-utility analysis						
Participants, No.	25	23	43	20	20	33
Cost per person, CAD$						
Mean (SD)	2161 (742)	3211 (1908)	2193 (1162)	3149 (1448)	4847 (3438)	3481 (1984)
Difference	−32 (258)	1018 (378)	0 [Reference]	−333 (511)	1366 (743)	0 [Reference]
QALY, mean (SD)	0.378 (0.043)	0.389 (0.055)	0.370 (0.049)	0.374 (0.050)	0.384 (0.061)	0.373 (0.056)
Adjusted QALY^b^						
Mean (SD)	0.381 (0.033)	0.382 (0.042)	0.372 (0.030)	0.376 (0.030)	0.379 (0.045)	0.374 (0.028)
Difference	0.009 (0.008)	0.010 (0.009)	0 [Reference]	0.002 (0.008)	0.004 (0.010)	0 [Reference]
ICUR	−3381^a^	101 687 (14 589 to −140 938)^c^	0 [Reference]	−154 198^a^	331 306 (−11 551 to −78 678)^d^	0 [Reference]

Abbreviations: ICER, incremental cost-effectiveness ratio; ICUR, incremental cost-utility ratio; NA, not applicable; QALY, quality-adjusted life-year.

^a^ There is no willingness-to-pay threshold for which we can be 95% confident that the 2 therapies differed in value.

^b^ QALYs are adjusted for baseline utility using a linear regression model.

^c^ Expressed as estimate (95% CI), with 95% CIs indicating that for willingness-to-pay threshold of at least CAD $0 and no more than CAD $14 589, we can be 95% confident that the therapy with the larger point estimate for effect represents good value compared with the alternative.

^d^ Expressed as estimate (95% CI), with 95% CIs indicating that there is no willingness-to-pay threshold for which we can be 95% confident that the 2 therapies differ in value.

### QALYs

The mean (SD) adjusted incremental QALYs at the end of the 6-month intervention was 0.009 (0.008) QALYs for exercise vs balance and tone groups and 0.010 (0.009) QALYs for cognitive and social enrichment vs balance and tone groups. The mean (SD) adjusted incremental QALYs after follow-up was 0.002 (0.008) years for exercise vs balance and tone groups and 0.004 (0.010) QALYs for cognitive and social enrichment vs balance and tone groups. QALYs were adjusted using a regression model that considered baseline utility and group.

### Cost-Effectiveness Analysis

In the base case analysis, the exercise intervention was costlier and more effective than the balance and tone intervention at the end of the 6-month intervention, with an ICER of CAD −$8823 (US −$6352.56) per mean change in ADAS-Cog-Plus score.

**Figure 1.**
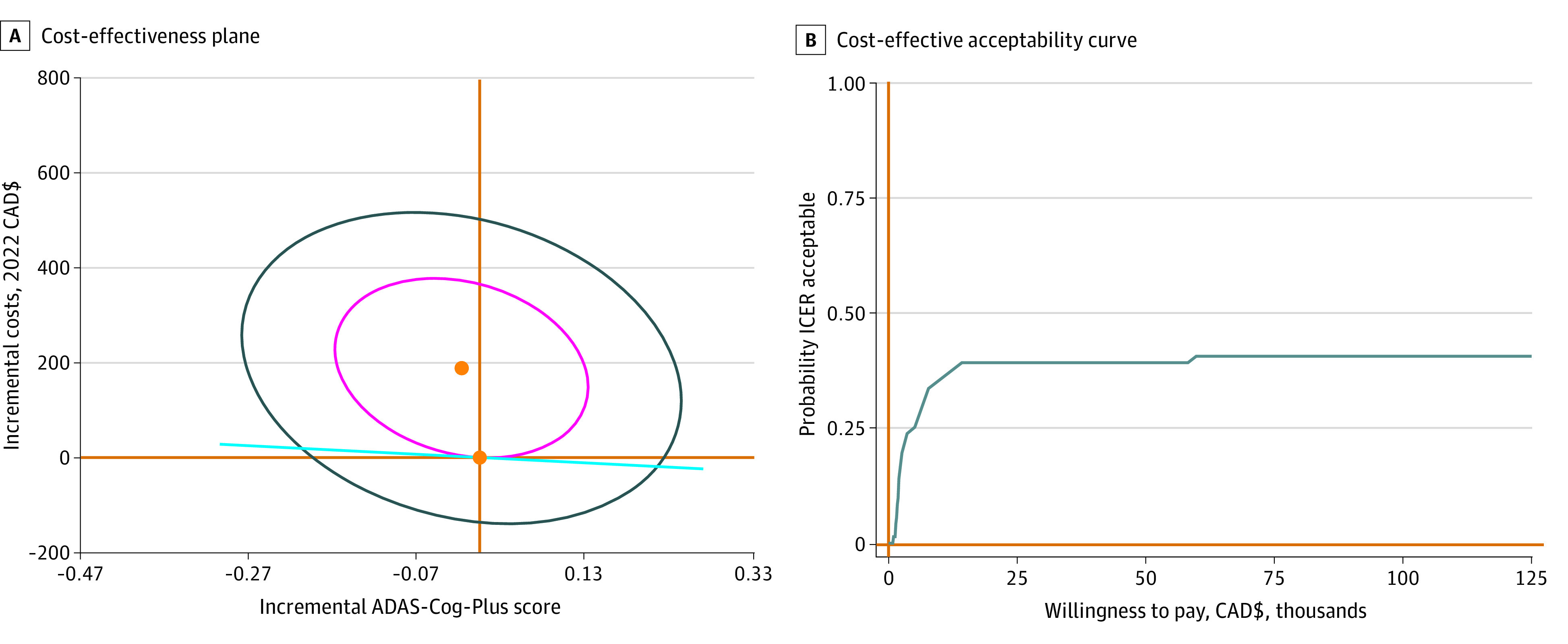
Cost-Effectiveness of Exercise Program vs Balance and Tone Program at 6 Months A. Orange lines indicate 0 on the x and y axes; orange dots, incremental cost effectiveness ratio estimates; light blue line and circles, 5000 bootstrapped estimates. B. Gray line indicates probability of incremental cost effectiveness ratio acceptance for different willingness-to-pay thresholds. ADAS-Cog-Plus indicates Alzheimer Disease Assessment Scale–Cognitive-Plus; ICER, incremental cost-effectiveness ratio.

Figure 1A shows that for the exercise group compared with the balance and tone group, approximately 49% of 5000 bootstrapped cycles fell in the northwest quadrant at the end of the intervention. Since negative ADAS-Cog-Plus scores indicate healthy cognitive functioning, the intervention was costlier and more effective at the end. The proportion of bootstrapped cycles within the cost-effective region implies a degree of uncertainty regarding the effects of exercise on both costs and health benefits. Figure 1B shows that for exercise vs balance and tone interventions, the probability of ICER acceptance for willingness-to-pay values between CAD $15 000 and $60 000 (US $10 800-$43 200) was approximately 40% at the end of the intervention.

### Cost-Utility Analysis

In the base case analysis, the exercise program was cost saving (ie, less costly and more effective) compared with the balance and tone program, with an ICER CAD −$3381 (US $2434.32) per QALY gained at the end of the intervention and an ICER of CAD −$154 198 (US $111 022.56) per QALY gained at the end of the follow-up period. The cognitive and social enrichment program was costlier and more effective compared with the balance and tone program, with an ICER of CAD $101 687 (95% CI, $14 589 to −$140 938; US $73 214.64 [95% CI, $10 504.08 to −$101 475.36]) per QALY gained at the end of intervention and an ICER of CAD $331 306 (95% CI, −$11 551 to −$78 678; US $238 540.32 [95% CI, −$8316.72 to −$56 648.16]) per QALY gained at the end of the follow-up period.

**Figure 2.**
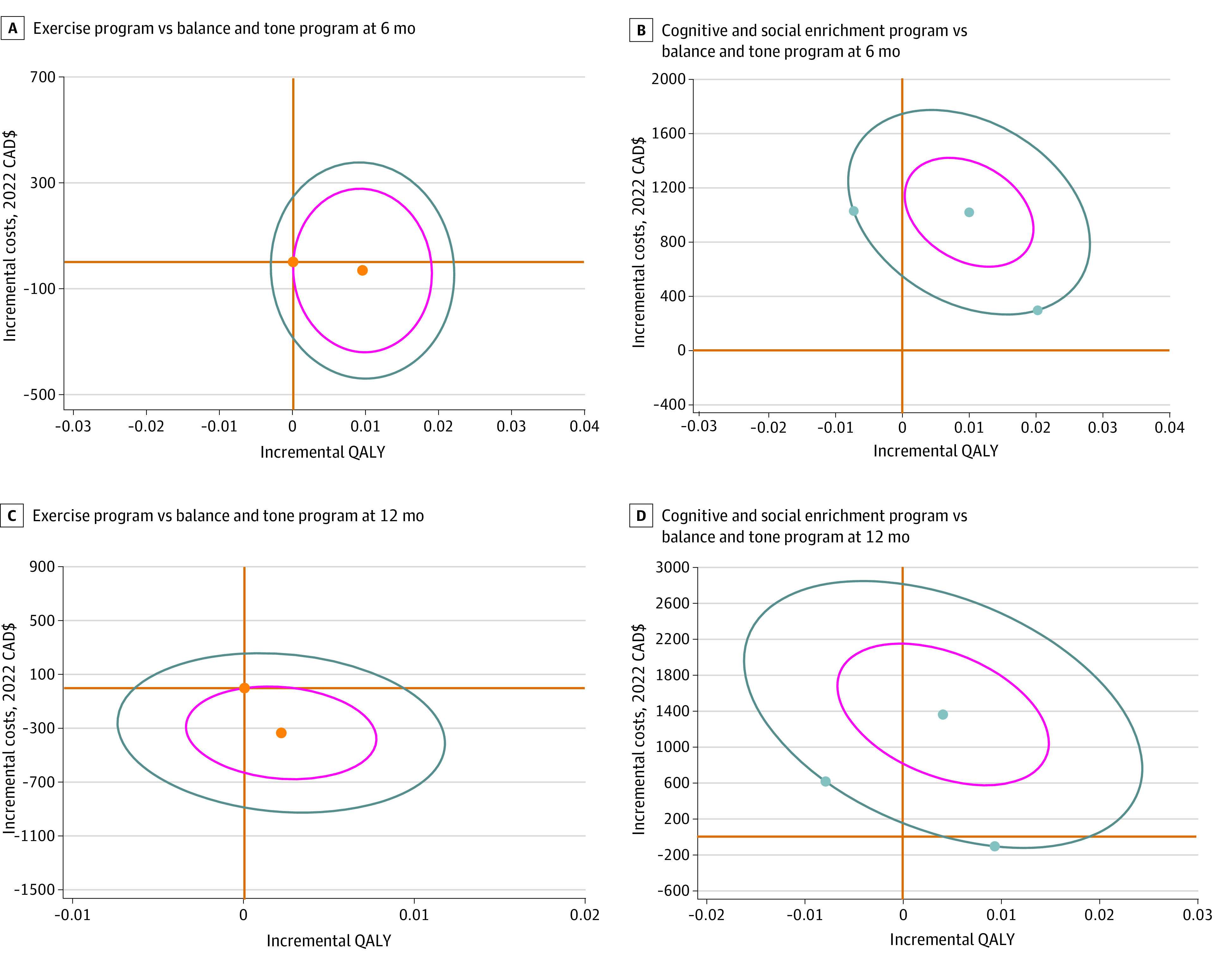
Cost Utility of Exercise Program and Cognitive and Social Enrichment Program vs Balance and Tone Program at 6 and 12 Months Thick orange lines indicate 0 on the x and y axes; orange dots, incremental cost effectiveness ratio estimates; circles, 5000 bootstrapped estimates; QALY, quality-adjusted life-year.

Figure 2A and C show that for the exercise intervention compared with the balance and tone intervention, most bootstrapped cycles (77%) fell in the southeast quadrant at the end of the intervention, and 50% of cycles fell in the northeast quadrant at the end of follow-up, indicating lower costs and higher effectiveness. Figure 2B and D show that for the cognitive and social enrichment intervention compared with the balance and tone intervention, approximately 99% of bootstrapped cycles fell in the northeast quadrant at the end of the intervention, and 96% of cycles fell in the northeast quadrant at the end of follow-up, indicating higher costs and lower effectiveness. The cost-effectiveness acceptability curve (eFigure in Supplement 1) demonstrates that both the exercise and cognitive and social enrichment programs had a 100% probability of ICUR acceptance when using the Canadian willingness-to-pay threshold of CAD $50 000 per QALY gained compared with the balance and tone program. However, none of the changes in QALYs from either exercise or cognitive and social enrichment programs met the MCID threshold of 0.03.^40^

### Sensitivity and Subgroup Analyses

Exercise was cost-effective for improving cognitive function, but not quality of life. Subgroup analyses revealed cost savings for exercise in participants aged 80 years and older, as well as participants who had a hemorrhagic stroke. The sensitivity analysis using imputed data produced results consistent with the base case analysis, confirming that the exercise program, while costlier, was more effective than the balance and tone program. eTable 2, eTable 3, eTable 4 and the eAppendix in Supplement 1 provide detailed sensitivity and subgroup analysis results.

## Discussion

This economic evaluation using data from the Vitality study found that at the end of the 6-month intervention, the exercise program was more effective in improving cognitive function among older adults with chronic stroke at additional cost compared with the control balance and tone program. As demonstrated by the primary RCT, exercise led to significant cognitive improvements, surpassing the MCID of 3 points on the 13-item ADAS-Cog.^22,47^ Hence, exercise likely represents a cost-effective means of enhancing cognitive function among individuals with chronic stroke at the end of the intervention period. The dearth of research on the efficiency of interventions for enhancing cognitive function in patients with chronic stroke hinders the comparison of these findings with existing research.^48^ Previous studies have shown diverse results due to variations in populations and methods used. Results from a simulation study with 1000 healthy older adults found that a combined physical exercise and cognitive program was cost-effective in preventing dementia.^49^ Among older adults with cognitive impairment, exercise interventions showed mixed results in terms of cost-effectiveness.^50^ Exercise was cost-effective for older adults with mild cognitive impairment but not for those with dementia.^51,52^

The cost-utility analysis demonstrated that neither exercise nor cognitive and social enrichment activities led to changes in health-related quality of life at the end of the intervention and follow-up. This study was powered to detect changes in cognitive function rather than quality of life; therefore, it lacked statistical power to detect differences in quality of life. Additionally, the limited responsiveness of the EQ-5D-3L might not capture condition-specific changes in quality of life.^21,53^

The social and cognitive enrichment intervention had higher delivery costs, and participants use more health care resources. Exercise, despite being more costly to deliver than the balance and tone program, led to the lowest health care resource utilization. Participants in the exercise group had lower health care costs for practitioner visits and laboratory tests. We posit that this may be attributed to exercise leading to improvement in cardiovascular fitness and overall health, resulting in reduced visits to practitioners.^54^

The exploratory subgroup analyses revealed that exercise was cost-saving for participants aged 80 years and older. This finding aligns with previous research that has demonstrated cost savings associated with exercise as a fall prevention strategy in older adults.^55^ Exercise was particularly beneficial for older adults who were more frail by improving their strength and balance to a level that enhances stability.^56^ For a population of adults with chronic stroke, this finding can be applied to underscore the importance of targeted interventions for older adults who are at a higher risk of cognitive decline.^5^

This study provides novel health economic data on the effectiveness of exercise for improving cognitive function in older adults with chronic stroke. Furthermore, since this was a trial-based economic evaluation, the study benefits from the design of the clinical trial, such as randomization and blinding, ensuring comparable data collection among intervention and control groups.^57,58^ Additionally, the trial’s use of established comparators, measurement of QALYs, sufficient follow-up period, adequate statistical power, and inclusion of a representative patient sample enhances its relevance for informing policy decisions.^59^

### Limitations

This study has limitations. The time horizon of the economic evaluation may not capture the longer-term costs and benefits of the interventions.^60^ The lack of established ADAS-Cog-Plus MCID and condition-specific willing-to-pay thresholds hampers cost-effectiveness comparisons across different conditions.^61^ The lack of stroke severity data limits our comprehension of diverse responses to interventions. The sample size, determined by the trial’s primary outcome, may introduce uncertainty in cost-effectiveness assessment. The comparator was not a null control approach, possibly leading to conservative intervention cost estimates. Self-reported health care resource use and potential recall bias could affect cost accuracy. Including proxy measures, like caregiver ratings, could have enhanced our study by providing additional information on health-related quality of life. This economic evaluation utilized a Canadian health care system perspective; thus, results may not be generalizable to health care systems outside of Canada.

## Conclusions

This economic evaluation provides novel evidence supporting the potential for multicomponent exercise as a cost-effective intervention for improving cognitive function in older adults with chronic stroke and reducing the economic burden of stroke. Cognitive and social enrichment activities show promise for improving cognitive function after stroke but come with higher costs. Future research should focus on optimizing the cost-effectiveness of these interventions and enhancing the health-related quality of life for this population.

## Notes

Supplement 1. **eAppendix.** Supplemental Materials **eTable 1.** Number and Proportion of Participants With Complete Data in the Vitality Study **eTable 2.** Results of Sensitivity Analyses Using Multiple Imputations **eTable 3.** Results of Subgroup Analyses for Cost-Effectiveness and Cost-Utility Analyses at 6 Months **eTable 4.** Results of Subgroup Analyses for Cost-Effectiveness and Cost-Utility Analyses at 12 Months **eFigure.** Cost-Effective Acceptability Curve for Cost-Utility Analysis at 6 Months and 12 Months

Supplement 2.
Data Sharing Statement
