# Supplementary material for: Economic Evaluation of Exercise or Cognitive and Social Enrichment Activities for Improved Cognition After Stroke
Source: JAMA Netw Open. 2023 Nov 30;6(11):e2345687. doi: 10.1001/jamanetworkopen.2023.45687 (PMC10690466; doi:10.1001/jamanetworkopen.2023.45687)
Supplement: Supplement 1. — eAppendix. Supplemental Materials eTable 1. Number and Proportion of Participants With Complete Data in the Vitality Study eTable 2. Results of Sensitivity Analyses Using Multiple Imputations eTable 3. Results of Subgroup Analyses for Cost-Effectiveness and Cost-Utility Analyses at 6 Months eTable 4. Results of Subgroup Analyses for Cost-Effectiveness and Cost-Utility Analyses at 12 Months eFigure. Cost-Effective Acceptability Curve for Cost-Utility Analysis at 6 Months and 12 Months [file jamanetwopen-e2345687-s001.pdf]

Supplemental Online Content

Adjetey C, Davis JC, Falck RS, et al. Economic evaluation of exercise or cognitive and social enrichment activities for improved cognition after stroke. *JAMA Netw Open*. 2023;6(11):e2345687. doi:10.1001/jamanetworkopen.2023.45687

eAppendix. Supplemental Materials

eTable 1. Number and Proportion of Participants With Complete Data in the Vitality Study

eTable 2. Results of Sensitivity Analyses Using Multiple Imputations

eTable 3. Results of Subgroup Analyses for Cost-Effectiveness and Cost-Utility Analyses at 6 Months

eTable 4. Results of Subgroup Analyses for Cost-Effectiveness and Cost-Utility Analyses at 12 Months

eFigure. Cost-Effective Acceptability Curve for Cost-Utility Analysis at 6 Months and 12 Months

© 2023 Adjetey C et al. *JAMA Network Open.*

eAppendix. Supplemental Material

A. SENSITIVITY ANALYSES- MULTIPLE IMPUTATION

Missing data remains a common problem in cost-effectiveness analyses due to issues relating to incomplete or non-responses on patient questionnaires, as well as participant dropout or withdrawal from a study.[1,2] A consort flow diagram showing the movement of participants through the trial is provided in Figures 1 and 2 below. A breakdown is provided for the cost-effectiveness analysis for participants with complete costs and ADAS-Cog-Plus score data only (Figure 1) and for the cost-utility analysis for participants with complete costs and QALY data only (Figure 2).

When missing data is not handled appropriately, this may lead to biased results and findings from cost-effectiveness analysis.[1,3] The method to address missing data is informed by the assumption of the missing data mechanism.[4] Missing data may be classified as missing completely at random (MCAR), missing at random (MAR), and missing not at random (MNAR). MCAR describes a mechanism where neither the missing data is independent of observed nor unobserved factors.[5] MAR describes a mechanism where missingness is unrelated to the unobserved values. [5] MNAR describes a mechanism where the unobserved variable itself predicts missingness.[5] While is it difficult to examine if MAR and MNAR hold for a particular dataset, it is possible to investigate if the assumption of MCAR holds.[1] For instance, data is unlikely to be MCAR if missing data differs by treatment group or baseline variables predict missingness.[1] We examined associations between baseline variables and missing data for the outcomes of interests using logic regression presented in Table 1.

MAR data mechanism was assumed and multiple imputation was used to predict estimates for missing costs and effectiveness data.[1,4,6,7] Multiple involves replacing missing values with plausible imputed datasets that are combined to account for uncertainty about the missing data.[8]

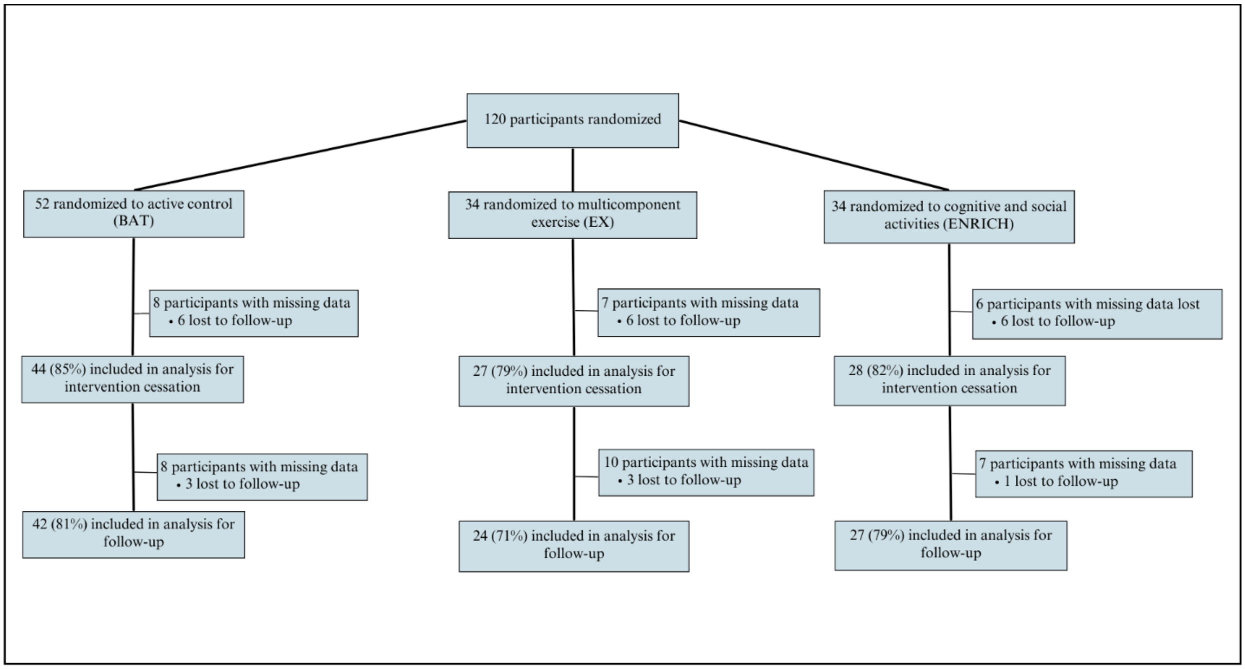

Figure 1. Consort flow diagram for cost-effectiveness analysis, adapted from the Vitality primary paper.[9]

© 2023 Adjetey C et al. *JAMA Network Open.*

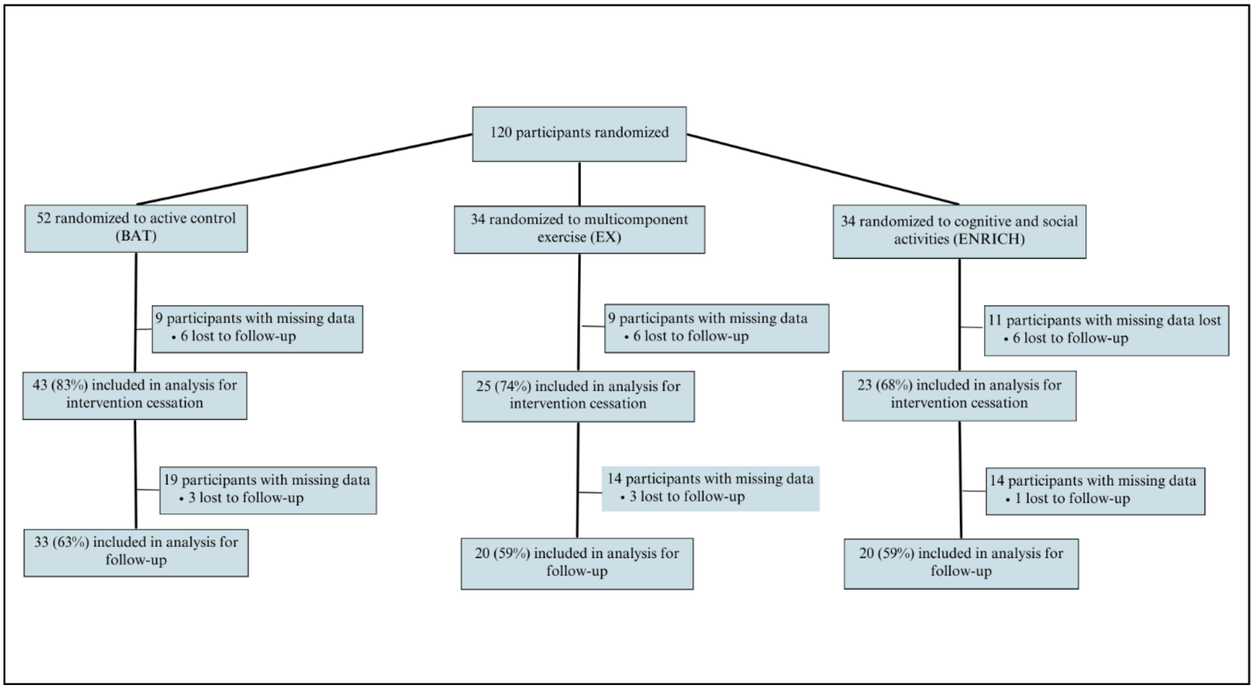

Figure 2. Consort flow diagram for cost-utility analysis, adapted from the Vitality primary paper.[9]

© 2023 Adjetey C et al. *JAMA Network Open.*

Table 1. Logistic regression for missingness of costs, quality-adjusted life-years on baseline variables

	End of intervention			End of follow up		
Baseline variable	Cost	QALY	ADAS-Cog-Plus	Cost	QALY	ADAS-Cog-Plus
Group	0.467 (0.440)	0.135 (0.336)	0.140 (0.422)	0.0795 (0.390)	0.0826 (0.272)	-0.0163 (0.415)
Functional Comorbidity Index	0.664* (0.262)	0.424* (0.177)	0.470* (0.213)	0.616** (0.217)	0.245 (0.136)	0.448* (0.201)
Age	0.0998 (0.0539)	0.0272 (0.0359)	0.0605 (0.0481)	0.0687 (0.0409)	-0.0124 (0.0291)	0.0800 (0.0438)
Male	-0.484 (1.069)	0.780 (0.894)	-0.105 (1.043)	0.999 (0.927)	-0.0988 (0.656)	1.381 (0.955)
Type of stroke: Ischemic	-0.533 (0.869)	0.527 (0.711)	-0.472 (0.782)	-0.280 (0.766)	0.00165 (0.512)	-0.315 (0.783)
Lacunar	0.521 (1.419)	2.980* (1.204)	-0.177 (1.553)	-0.0204 (1.357)	-0.0662 (0.979)	-0.813 (1.544)
Unknown	1.621 (1.549)	1.012 (1.176)	-0.478 (1.764)	0.239 (1.548)	-0.350 (1.051)	-0.234 (1.536)
Montreal Cognitive Assessment	0.227 (0.160)	0.137 (0.124)	0.179 (0.154)	0.438** (0.160)	-0.0417 (0.0902)	0.291 (0.158)
Center for Epidemiological Studies - Depression Scale	-0.0684 (0.0825)	0.0000734 (0.0448)	-0.0546 (0.0705)	0.0233 (0.0557)	-0.0218 (0.0328)	0.124* (0.0570)
Instrumental Activities of Daily Living	-0.331 (0.268)	-0.263 (0.203)	-0.00360 (0.248)	-0.419 (0.239)	-0.194 (0.167)	-0.346 (0.267)
Alzheimer's Disease Assessment Scale-Cognitive Plus	1.920* (0.901)	0.387 (0.589)	1.617* (0.767)	1.824* (0.765)	-0.00525 (0.452)	1.252 (0.695)
Weight(kg)	0.216 (0.290)	-0.176 (0.185)	0.0561 (0.239)	-0.116 (0.237)	0.0347 (0.133)	-0.0949 (0.240)
Body Mass Index	-0.542 (0.802)	0.396 (0.493)	-0.0837 (0.660)	0.206 (0.618)	-0.136 (0.358)	0.209 (0.627)
Six Minute Walk Test	-0.0018 (0.006)	-0.0023 (0.004)	-0.0003 (0.006)	-0.0056 (0.005)	-0.0053 (0.004)	-0.0096 (0.006)
Gait speed	0.233 (2.199)	-0.261 (1.652)	1.415 (2.096)	-0.0262 (1.945)	1.499 (1.292)	0.173 (1.985)
Height	-0.142 (0.262)	0.171 (0.172)	-0.004(0.216)	0.088 (0.214)	-0.0304 (0.124)	0.136 (0.216)
Mini-Mental State Examination	-0.345 (0.212)	-0.171 (0.168)	-0.225 (0.203)	-0.456* (0.216)	-0.102 (0.141)	-0.489* (0.223)
Short Physical Performance Battery	-0.108 (0.296)	-0.118 (0.226)	-0.185 (0.285)	-0.0632 (0.281)	-0.0955 (0.179)	0.182 (0.268)
Self-efficacy	0.0005 (0.002)	0.0014 (0.0013)	-0.0006 (0.0015)	0.0019 (0.001)	0.0014 (0.0009)	0.0024 (0.0015)
Physical Activity (caloric expenditure/wk in all exercise-related activities)	-0.000598* (0.000303)	-0.0000764 (0.000148)	-0.000429 (0.000253)	-0.000480 (0.000257)	-0.000146 (0.000114)	-0.000774* (0.000325)
EQ-5D-3L	1.458 (4.036)	4.090 (3.646)	-0.760 (3.885)	-1.005 (3.661)	0.987 (2.227)	3.428 (3.996)
Constant	17.16 (44.15)	-31.49 (29.38)	-4.343 (37.28)	-13.79 (35.90)	10.11 (21.80)	-24.99(36.66)
Observations	117	117	117	117	117	117

Indicates statistical significance * $p < 0.05$, ** $p < 0.01$, *** $p < 0.001$
Standard errors in parentheses

© 2023 Adjetey C et al. *JAMA Network Open.*

A.1 Multiple Imputation model

We used Multiple Imputation by Chained Equation (MICE) in STATA to address missing data by specifying one imputation model for each variable of interest (i.e. costs, ADAS-Cog-Plus, and Quality Adjusted Life Years (QALYs)). Five-thousand iterations of bootstrapping were used to quantify uncertainty and determine which values were more likely.[10]

Here is a sample specification of our multiple imputation model as well as a brief description of each of the components:

mi impute chained (pmm, knn(5)) missing data variable*)* = complete baseline variables, Add (Number of imputations), by treatment group, set seed, where:

a) Mi impute chained represents “multiple imputation using chained equations”.
b) Pmm(knn) represents “predictive mean matching”. This ensured that the imputed values for missing costs and effectiveness outcomes were in an acceptable range.1
c) Missing data variable represents either one of cost, ADAS-Cog-Plus and QALY variables with missing data
d) Complete baseline variable represents auxiliary variables that were found to be associated with missingness based on results from the logistic regression presented in Table 1.
e) Add (number of imputation) represented the number of imputations specified in our model which was 50.
f) By treatment group ensured that the values were imputed by different treatment groups.

© 2023 Adjetey C et al. *JAMA Network Open.*

eTable 1. Number and proportion of participants with complete data in Vitality study

Variable	All participants (n=120)	Exercise (n=34)	Cognitive and social enrichment (n=34)	Balance and tone (n=52)
	Number (%)			
ADAS-COG-PLUS baseline	120 (100%)	34 (100%)	34 (100%)	52 (100%)
ADAS-COG-PLUS endpoint	103 (86%)	29 (85%)	29 (85%)	45 (87%)
ADAS-COG-PLUS follow-up	96 (80%)	25(74%)	28 (82%)	43 (83%)
EQ-5D-3L at timepoint 1 - baseline	120 (100%)	34 (100%)	34 (100%)	52 (100%)
EQ-5D-3L at timepoint 2	107 (89%)	30 (88%)	30 (88%)	47 (90%)
EQ-5D-3L at timepoint 3	105 (88%)	30 (88%)	29 (85%)	46 (89%)
EQ-5D-3L at timepoint 4	104 (87%)	29 (85%)	29 (85%)	46 (89%)
EQ-5D-3L at timepoint 5	101 (84%)	29 (85%)	26 (77%)	46 (89%)
EQ-5D-3L at timepoint 6	102 (85%)	29 (85%)	28 (82%)	45 (87%)
EQ-5D-3L at timepoint 7 – 6 months	100 (83%)	27 (79%)	29 (85%)	44 (85%)
EQ-5D-3L at timepoint 8	100 (83%)	29 (85%)	27 (79%)	44 (85%)
EQ-5D-3L at timepoint 9	99 (83%)	28 (82%)	28 (82%)	43 (83%)
EQ-5D-3L at timepoint 10	99 (83%)	29 (85%)	27 (79%)	43 (83%)
EQ-5D-3L at timepoint 11	96 (80%)	28 (82%)	27 (79%)	41 (79%)
EQ-5D-3L at timepoint 12	95 (79%)	27 (79%)	27 (79%)	41 (79%)
EQ-5D-3L at timepoint 13 – follow up	87 (72%)	25 (74%)	24 (71%)	38 (73%)
All 13 timepoints	73 (61%)	20 (59%)	19 (56%)	34 (65%)
QALY at end of intervention	93 (78%)	25 (74%)	24 (71%)	44 (85%)
QALY at follow up	77 (64%)	22 (65%)	21 (62%)	34 (65%)
HRU- timepoint 1 baseline	119 (99%)	34 (100%)	34 (100%)	51 (98%)
HRU- timepoint 4 midpoint	107 (89%)	30 (88%)	30 (88%)	47 (90%)
HRU- timepoint 7 endpoint	101 (84%)	27 (79%)	28 (82%)	46 (89%)
HRU- timepoint 13 follow up	98 (82%)	26 (77%)	29 (85%)	43 (83%)
HRU- Total at end of intervention	100 (83%)	27 (79%)	28 (82%)	45 (87%)
HRU- Total at follow up	95 (79%)	25 (74%)	28 (82%)	42 (81%)

© 2023 Adjetey C et al. *JAMA Network Open.*

eTable 2. Results of sensitivity analyses

Scenario	Outcomes	Intervention cessation (at 6 months)			End of follow-up (at 12 months)		
		Exercise	Cognitive and social enrichment	Balance and tone	Exercise	Cognitive and social enrichment	Balance and tone
Multiple imputations	Number of participants	N = 34	N = 34	N = 52	N = 34	N = 34	N = 52
	Mean (SD) cost	2250 (192)	3310 (347)	2130 (152)	3283 (352)	4685 (568)	3253 (245)
	Incremental cost	120 (33)	1178 (45)	Reference	32 (56)	1429 (73)	Reference
	ADAS-Cog-Plus score	-0.080 (0.146)	NA	-0.043 (0.138)	NA	NA	-0.109 (0.140)
	Incremental ADAS-Cog-Plus	-0.036 (0.233)	NA	Reference	NA	NA	Reference
	Incremental Cost/ ADAS-Cog-Plus	-3308 [95% CI: -1122, 5038]	NA	Reference	339 [95% CI: -790, 2340]	NA	Reference
	QALY	0.381 (0.005)	0.380 (0.006)	0.376 (0.004)	0.372 (0.004)	0.377 (0.005)	0.379 (0.003)
	Incremental QALY	0.005 (0.001)	0.005 (0.001)	Reference	-0.007 (0.001)	-0.002 (0.001)	Reference
	Incremental Cost/ QALY	24300 [95% CI:10519, 44122]	255253 [95% CI:179888, 421361]	Reference	-4409 [95% CI: -19632, 10934]	-804739 [95% CI: -5914400, -438911]	Reference
Baseline adjusted ADAS-Cog-Plus score	Adjusted ADAS-Cog-Plus	-0.175 (0.682)	-0.216 (0.683)	-0.161 (0.814)	-0.107 (0.670)	-0.180 (0.691)	-0.258 (0.818)
	Incremental Adjusted ADAS-Cog-Plus	-0.0148 (0.187)	NA	Reference	NA	NA	NA
Total costs assuming intervention costs includes only personnel costs	Mean (SD) cost	2041 (1188)	2508 (1973)	2126 (1150)	3219 (2260)	3918 (3253)	3235 (1826)
	Incremental cost	-85 (285)	383 (368)	Reference [a]	-16 (510)	682 (612)	Reference [a]
	Incremental ADAS-Cog-Plus	-0.021 (0.225)	NA	Reference [a]	NA	NA	Reference [a]
	Incremental Cost/ ADAS-Cog-Plus	3992	NA	Reference [a]	NA	NA	Reference [a]
	Incremental cost	-305 (259)	344 (378)	Reference [a]	-605 (510)	691 (743)	Reference [a]
	Incremental QALY	0.009 (0.008)	0.010 (0.009)	Reference [a]	0.002 (0.008)	0.004 (0.010)	Reference [a]
	Incremental Cost/ QALY	-32109	34338	Reference [a]	-280535	167711	Reference [a]
Total costs including healthcare resource utilization only	Mean (SD) cost	1261 (1188)	1728 (1973)	1386 (1150)	2439 (2260)	3138 (3253)	2496 (1826)
	Incremental cost	-125.39 (284.70)	342 (368)	Reference [a]	-57 (510)	642 (612)	Reference [a]
	Incremental ADAS-Cog-Plus	-0.021 (0.225)	NA	Reference [a]	NA	NA	Reference [a]
	Incremental Cost/ ADAS-Cog-Plus	5897	NA	Reference [a]	-369	NA	Reference [a]

© 2023 Adjetey C et al. *JAMA Network Open.*

	Outcomes	Intervention cessation (at 6 months)			End of follow-up (at 12 months)		
		**Exercise**	**Cognitive and social enrichment**	**Balance and tone**	**Exercise**	**Cognitive and social enrichment**	**Balance and tone**
	Incremental cost	-345 (259)	303 (378)	Reference [a]	-646 (511)	651 (742)	Reference [a]
	Incremental QALY	0.009 (0.008)	0.010 (0.009)	Reference [a]	0.002 (0.008)	0.004 (0.010)	Reference [a]
	Incremental Cost/ QALY	-36378	30294	Reference [a]	-299312	157888	Reference [a]

[a] Reference indicates that the comparator is the stretching and toning (control) group.
[b] There is no WTP for which we can be 95% confident that the two therapies differ in value.
[c] QALYs are adjusted for baseline utility using a linear regression model.
[d] Confidence interval is written as [lower limit, upper limit] and interpreted as: for WTP ≥ 0 & ≤ 14589: We can be 95% confident that the therapy with the larger point estimate for effect represents good value compared with the alternative.
[e] Confidence interval is written as [lower limit, upper limit] and interpreted as: there is no WTP for which we can be 95% confident that the 2 therapies differ in value.

© 2023 Adjetey C et al. *JAMA Network Open.*

eTable 3. Results of subgroup analyses for cost-effectiveness and cost-utility analyses at 6 months

Outcomes	Subgroup	N	EX vs BAT	ENRICH vs BAT
	ADAS-Cog-Plus at baseline			
Incremental costs	Greater than or equal to 0	56	147 (184)	NA
Incremental ADAS-Cog-Plus			-0.20 (0.22)	
Incremental Cost/ ADAS-Cog-Plus			-738*	
Incremental costs		54	91 (191)	311 (166)
Incremental QALY			0.015 (0.011)	0.030 (0.009)
Incremental Cost/ QALY			6232*	10237
Incremental costs	Less than 0	43	320 (593)	NA
Incremental ADAS-Cog-Plus			0.076 (0.218)	
Incremental Cost/ ADAS-Cog-Plus			4204*	
Incremental costs		37	-185.53 (552.52)	2166.33 (763.22)
Incremental QALY			0.002 (0.011)	-0.021 (0.016)
Incremental Cost/ QALY			-86039*	-103149 [132327, -24205]
	Age			
Incremental costs	Greater than or equal to 80	17	-173 (212)	NA
Incremental ADAS-Cog-Plus			-0.059 (0.528)	
Incremental Cost/ ADAS-Cog-Plus			2929*	
Incremental costs		16	-310 (221)	184 (271)
Incremental QALY			0.025 (0.013)	0.023 (0.017)
Incremental Cost/ QALY			-12449*	8010 (-10515, -33519]
Incremental costs	Less than 80	82	279 (341)	NA
Incremental ADAS-Cog-Plus			-0.120 (0.240)	
Incremental Cost/ ADAS-Cog-Plus			-2321*	
Incremental costs		75	26 (310)	1236 (453)
Incremental QALY			0.006 (0.009)	0.007 (0.011)
Incremental Cost/ QALY			4375*	179491 [19586, -90053]
	Type of stroke			
Incremental costs	Hemorrhagic stroke	27	-185 (329)	NA
Incremental ADAS-Cog-Plus			0.497 (0.453)	
Incremental Cost/ ADAS-Cog-Plus			-372*	
Incremental costs		28	-247 (326)	6423 (385)
Incremental QALY			0.001 (0.020)	0.024 (0.014)
Incremental Cost/ QALY			-369564	26828

© 2023 Adjetey C et al. *JAMA Network Open.*

Outcomes	Subgroup	N	EX vs BAT	ENRICH vs BAT
Incremental costs	Ischemic & Lacunar stroke	72	312 (362)	NA
Incremental ADAS-Cog-Plus			-0.207 (0.260)	
Incremental Cost/ ADAS-Cog-Plus			-1507*	
Incremental costs		63	42 (336)	1241 (523)
Incremental QALY			0.013 (0.008)	0.002 (0.116)
Incremental Cost/ QALY			3213*	498712 (14683, -53848]

* There is no Willingness-to-pay (WTP) for which we can be 95% confident that the two therapies differ in value.

© 2023 Adjetey C et al. *JAMA Network Open.*

eTable 4. Results of subgroup analyses for cost-utility analyses at 12 months

Outcomes	Subgroup	N	EX vs BAT	ENRICH vs BAT
	ADAS-Cog-Plus at baseline			
Incremental costs	Greater than or equal to 0	42	88.41 (453.52)	2.82 (420.30)
Incremental QALY			0.003 (0.012)	0.022 (0.001)
Incremental Cost/ QALY			28424*	126 [-28066, 291012]
Incremental costs	Less than 0	31	-707.07 (1055.27)	3634.96 (1312.89)
Incremental QALY			0.001 (0.012)	-0.023 (0.018)
Incremental Cost/ QALY			-495827*	-159149 [192103, -35481]
	Age			
Incremental costs	Greater than or equal to 80	15	-243.47 (649.57)	374.32 (788.38)
Incremental QALY			0.006 (0.01)	0.01 (0.16)
Incremental Cost/ QALY			-38160*	37054
Incremental costs	Less than 80	58	-300.84 (617.79)	1882.38 (914.24)
Incremental QALY			-0.0001 (0.01)	0.0001 (0.012)
Incremental Cost/ QALY			1507353*	12600429 [3665, -58851]
	Type of stroke			
Incremental costs	Hemorrhagic stroke	21	-1223.94 (784.57)	132.95 (1002.84)
Incremental QALY			-0.003 (0.023)	0.022 (0.016)
Incremental Cost/ QALY			351469*	5933
Incremental costs	Ischemic & Lacunar stroke	52	49.90 (639.23)	1894.89 (954.95)
Incremental QALY			0.005 (0.008)	-0.004 (0.0126)
Incremental Cost/ QALY			10851*	-513843 [-7863, -32059]

* There is no Willingness-to-pay (WTP) for which we can be 95% confident that the two therapies differ in value.

© 2023 Adjetey C et al. *JAMA Network Open.*

eFigure 1. Cost-effective acceptability curve for cost-utility analysis at 6 months and 12 months.

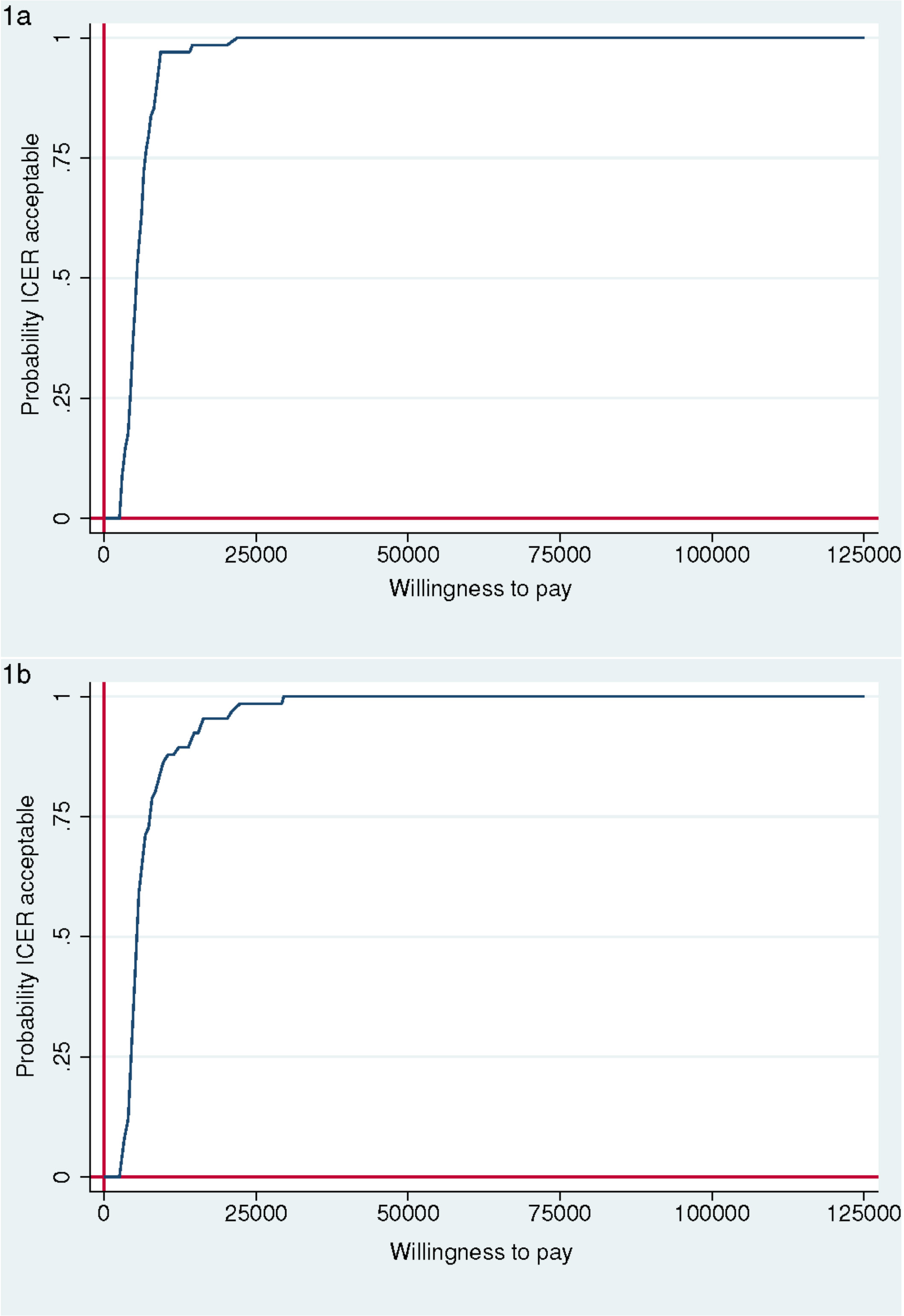

© 2023 Adjetey C et al. *JAMA Network Open.*

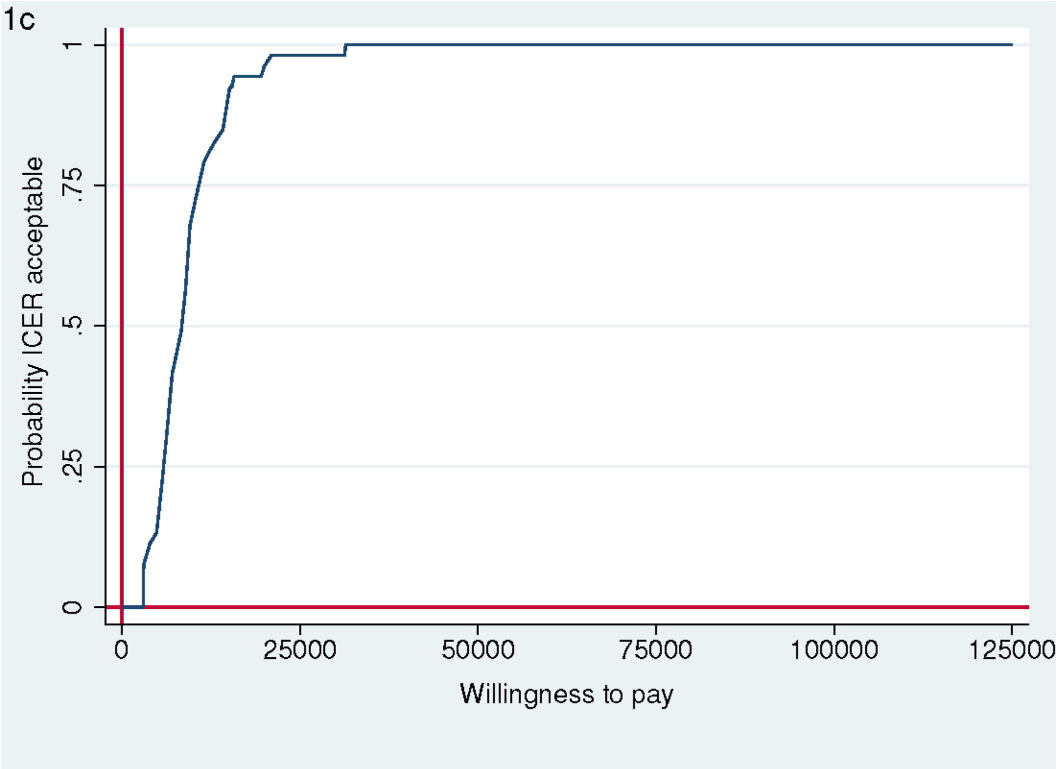

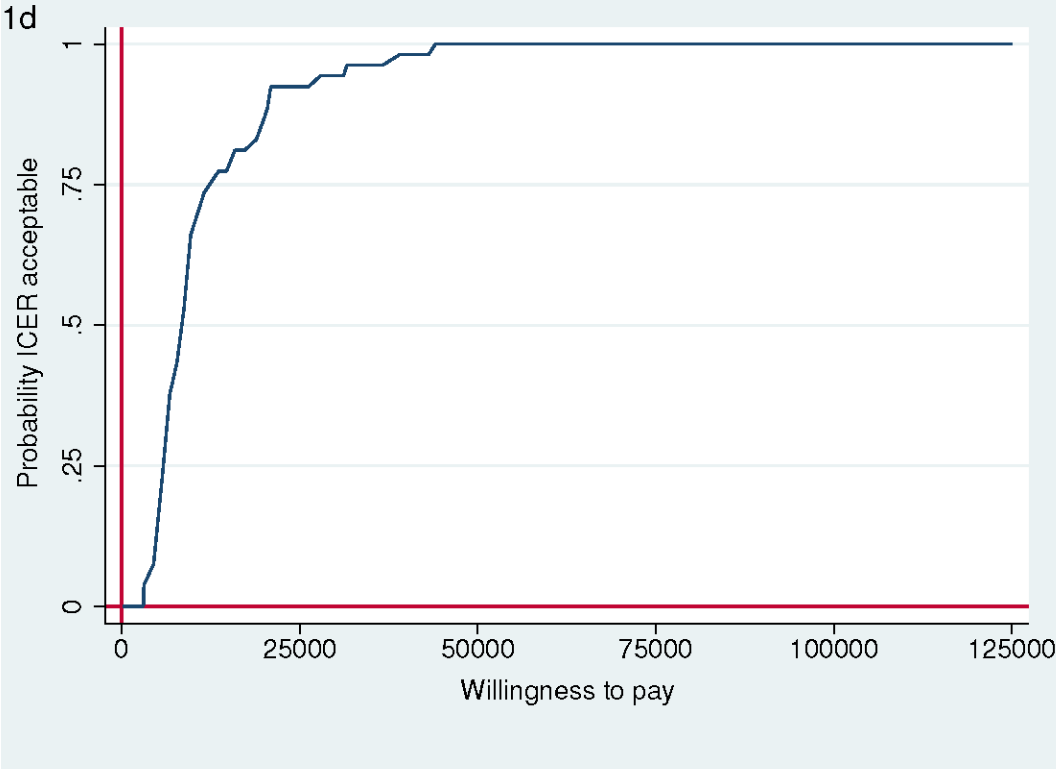

© 2023 Adjetey C et al. *JAMA Network Open.*

References

1. Faria R, Gomes M, Epstein D, White IR. A Guide to Handling Missing Data in Cost-Effectiveness Analysis Conducted Within Randomised Controlled Trials. *PharmacoEconomics*. 2014;32(12):1157-1170. doi:10.1007/s40273-014-0193-3

2. Petrou S, Gray A. Economic evaluation alongside randomised controlled trials: design, conduct, analysis, and reporting. *The BMJ*. 2011;342:d1548. doi:10.1136/bmj.d1548

3. Hughes D, Charles J, Dawoud D, et al. Conducting Economic Evaluations Alongside Randomised Trials: Current Methodological Issues and Novel Approaches. *PharmacoEconomics*. 2016;34(5):447-461. doi:10.1007/s40273-015-0371-y

4. Gabrio A, Mason AJ, Baio G. Handling Missing Data in Within-Trial Cost-Effectiveness Analysis: A Review with Future Recommendations. *PharmacoEconomics Open*. 2017;1(2):79-97. doi:10.1007/s41669-017-0015-6

5. Rubin D. Inference and missing data. *Biometrika*. 1976;63(3):581-592. doi:10.1093/biomet/63.3.581

6. Manca A, Palmer S. Handling missing data in patient-level cost-effectiveness analysis alongside randomised clinical trials. *Appl Health Econ Health Policy*. 2005;4(2):65-75. doi:10.2165/00148365-200504020-00001

7. Oostenbrink J, Al M, Rutten-van Mölken M. Methods to analyse cost data of patients who withdraw in a clinical trial setting. *PharmacoEconomics*. 2003;21(15):1103-1112. doi:10.2165/00019053-200321150-00004

8. Sterne JAC, White IR, Carlin JB, et al. Multiple imputation for missing data in epidemiological and clinical research: potential and pitfalls. *BMJ*. 2009;338:b2393. doi:10.1136/bmj.b2393

9. Liu-Ambrose T, Falck RS, Dao E, et al. Effect of Exercise Training or Complex Mental and Social Activities on Cognitive Function in Adults With Chronic Stroke: A Randomized Clinical Trial. *JAMA Netw Open*. 2022;5(10):e2236510. doi:10.1001/jamanetworkopen.2022.36510

10. Khan S, Ahmad A, Mihailidis A. Bootstrapping and Multiple Imputation Ensemble Approaches for Missing Data. *J Intell Fuzzy Syst*. Published online September 25, 2019.

© 2023 Adjetey C et al. *JAMA Network Open.*
