# Supplementary material for: Economic Evaluation of Exercise or Cognitive and Social Enrichment Activities for Improved Cognition After Stroke
Source: JAMA Netw Open. 2023 Nov 30;6(11):e2345687. doi: 10.1001/jamanetworkopen.2023.45687 (PMC10690466; doi:10.1001/jamanetworkopen.2023.45687)
Supplement: Supplement 2. — Data Sharing Statement [file jamanetwopen-e2345687-s002.pdf]

Data Sharing Statement

Adjetey. Economic Evaluation of Exercise or Cognitive and Social Enrichment Activities for Improved Cognition After Stroke. *JAMA Netw Open*. Published November 30, 2023. doi:10.1001/jamanetworkopen.2023.45687

Data
Data available: No

Additional Information
Explanation for why data not available: The University of British Columbia Ethics board requires that data requests be made directly to the principal investigator in order to process an ethics amendment.
